# Modern Medico-Psychology and Psychiatry

**Published:** 1895-01-12

**Authors:** T. S. Clouston

**Affiliations:** Lecturer on Mental Diseases, Edinburgh University, and Physician Superintendent Royal Asylum, Morningside


### MODERN MEDICO-PSYCHOLOGY AND PSYCHIATRY.

By T. S. CLOUSTON, M.D., Lecturer on Mental Diseases, Edinburgh University, and Physician Superintendent Royal Asylum, Morningside.

### XIV.—STATES OF DEFECTIVE INHIBITION AND OF UNCONTROLLABLE IMPULSE.

(*Continued from page* 201.)

THE forms assumed by morbid impulse and loss of control are innumerable in kind and most various in degree. They may result either from loss of the inhibitory power, or from an excessive and sudden output of energy in the brain centres that cannot be controlled or regulated by those centres. Both states are equally conditions of disease. I have now two patients who afford examples of the simplest forms of impulsive and uncontrollable insane acts. Those acts are the chief results and symptoms remaining after attacks of acute insanity, showing that such a brain storm in each case had left the controlling mental centres weakened and unfit to regulate the acts of the lower centres. The one indulges in vocal explosions at regular intervals of a few seconds, producing a sound resembling "ah," pronounced in a quick catchy way like a vocal sigh. When one speaks to her she is sensible and coherent, and stops the noise. When eating and walking she is silent. When asked why she makes this sound she says she cannot help it, and that making it gives her relief. It is the chief morbid symptom left of the former groaning and restlessness of the attack of excited melancholia under which she laboured for many months after coming into the asylum. Though she can stop it for a time by a special effort, or when her attention is taken up, she cannot do so for long. We are trying two modes of treatment, which are both applicable to all such cases. The one is dietetic and medical, by giving nourishing foods and nerve tonics, and so restoring the exhausted inhibitory cells of the brain centre as well as the general nourishment of the whole body. The second therapeutic method is to get her to put into action other motor centres and other muscles than those of the voice, and so to withdraw energy from the explosive centres. This we do by encouraging work, talking, walking, amusements, and social intercourse. If left alone she would go into a room by herself, lie on a bed, cover up her head, and go on uninterruptedly making her rhythmical explosive "ahs." The constant repetition of any motor or mental act or co-ordination tends to open up nerve paths in the brain, and so to make its repetition easier. In disease this repetition may become an automatic act, constantly repeated, and unstimulated by any volitional impulse at all. In the other case of this disease the woman rubs one thumb against the forefingers of the other hand in a rhythmical way all day, except when she is eating or sleeping or walking fast. This has gone on for many years. Every method has been tried to stop this morbid act of insane impulse, but without success. She can stop the motion for a minute or so if she is tremendously scolded, but the stoppage evidently causes her great uneasiness, which becomes more and more intolerable until she begins it again. We have tried to make her knit, walk, and polish floors, but the motor energy cannot be permanently drawn from these hand and arm centres, nor can the higher inhibitory centres be strengthened to stop it.

But it may be asked—What have the simple motor phenomena in these two cases to do with uncontrollable tendencies towards murder, theft, sexual crime, destructiveness, &c.? With our present knowledge of brain function, and our interpretation of the facts, all these tendencies and acts are of essentially the same nature as these two simple and perfectly harmless motor automatisms in those two women. Let us look at a case of homicidal impulse—to take the more serious of all the forms of insane "psychokinesia"—which was once under my care, and endeavour to see its real significance as a symptom of brain disorder. A man, J. R., who had been in the army in India, had sunstroke, and also had drunk hard. These two sources of brain disease had in him weakened not his intellect nor his affections specially, for he came home and set up a shop and made money, and he married a wife with whom he lived affectionately. But it weakened his power of control, and under certain circumstances set up violent homicidal impulses. The sunstroke and alcohol had in J. R. the same effect in damaging the power of his inhibitory centres as the previous attacks of acute insanity had had in the cases of the two women to whom I have referred. When J. R. got into a passion, that is, when an excitation into unusual action of large mental tracts in his brain took place, he lost not only his control over his actions, but over his reasoning and special sense centres, for he developed morbid unfounded suspicions, and hallucinations of hearing. When he took whisky, even in small quantities, which he very seldom did, that is, when a physical brain stimulant determined much blood to his brain and roused the cells into a still more active state, he would give way to most dangerous and quite uncontrollable homicidal impulses, which would continue after the liquor had got out of his brain. In one of those he stabbed and killed his wife, to whom he was much attached. When he did so he was scarcely conscious. It was not a purposive but a truly automatic act, unchecked by any higher "motive."

Suicide is constantly committed from morbidly lessened power of action in the inhibitory centres in the brain, or from a violent impulse to do the act of self-destruction, which goes against the strongest of all the natural instincts in every normally constituted animal, from the highest to the lowest. To non-medical minds it seems much easier to allow that an act of suicide may be the result of a morbid uncontrollable impulse, than an act of murder. In reality, both may be of precisely the same nature, and from the same cause, viz., disease of the controlling centres of the brain. Not a day passes but such an act of suicide is committed in the British Empire, many of the persons being popularly reckoned otherwise sane.

